# Hypokalemic Quadriparesis as the Initial Presentation of Primary Sjögren's Syndrome With Distal Renal Tubular Acidosis: A Case Report

**DOI:** 10.1002/ccr3.71630

**Published:** 2025-12-07

**Authors:** Sagun Baral, Sushmita Bhattarai, Ajaya Raj Gautam, Prabesh Raghubanshi, Krishna Kc

**Affiliations:** ^1^ Department of Internal Medicine Rapti Academy of Health Sciences Dang Nepal

**Keywords:** hypokalemia, paralysis, quadriparesis, renal tubular acidosis, Sjögren's syndrome

## Abstract

Primary Sjögren's syndrome (PSS) typically presents with sicca symptoms, while renal involvement, such as distal renal tubular acidosis (dRTA), is less common. Hypokalemic paralysis as the initial manifestation of PSS is rare. We report a 52‐year‐old woman who presented with acute flaccid quadriparesis due to severe hypokalemia and normal anion gap metabolic acidosis consistent with dRTA. Autoimmune workup revealed ANA, anti‐Ro/SSA, and anti‐La/SSB positivity with an abnormal Schirmer's test, establishing the diagnosis of PSS despite the absence of dryness symptoms. The patient improved rapidly with potassium and bicarbonate replacement. This case underscores the importance of considering PSS in patients with unexplained hypokalemic paralysis and metabolic acidosis, even in the absence of sicca features, as timely recognition prevents life‐threatening complications.

## Key Clinical Message

- Acute hypokalemic quadriparesis can be the initial manifestation of primary Sjögren's syndrome, secondary to underlying distal renal tubular acidosis (dRTA).
- The diagnosis of Sjögren's should be actively pursued with serological testing in patients with unexplained hypokalemia and metabolic acidosis, even if classic sicca symptoms are absent.

## 1 Introduction

Primary Sjögren's syndrome (PSS) is a common autoimmune exocrinopathy, with peak incidence around 50 years of age and a female‐to‐male ratio of 9:1 [1]. Immune‐mediated damage of glands results in xerophthalmia (dry eyes) and xerostomia (dry mouth). It may involve other mucosal surfaces such as airways, the digestive tract, and the vagina, leading to classical “sicca syndrome” [2]. Approximately 80% of patients present with the triad of dryness of mouth and eyes, fatigue, and joint pain, whereas 30%–40% of patients experience systemic complications [1]. Renal involvement is a rare manifestation, occurring in fewer than 10% of cases [3]. Renal involvement varies over a wide spectrum from isolated electrolyte disturbances to nephrolithiasis, glomerulonephritis, and tubulointerstitial nephritis (TIN). TIN secondary to lymphocytic infiltration of the renal interstitium is the most common renal manifestation [3]. Renal tubular dysfunction leads to acid retention or bicarbonate loss, manifesting as renal tubular acidosis (RTA) [4]. RTA may be classified as dRTA, when there is inadequate hydrogen ion excretion in the distal nephron, or proximal (Fanconi syndrome), when the proximal tubule is involved [5].

Hypokalemic quadriparesis as the initial manifestation of dRTA secondary to PSS is rare. We present a case of a 52‐year‐old woman with PSS whose initial manifestation was life‐threatening hypokalemic quadriparesis secondary to dRTA, highlighting the importance of considering this autoimmune diagnosis in patients with unexplained electrolyte disturbances, even when classic sicca symptoms are absent.

## 2 Case History/Examination

A 52‐year‐old woman with no known comorbidities presented to the emergency department with a 2‐day history of progressive, symmetric weakness in all four limbs, rendering her unable to rise from bed. The weakness was symmetric and more prominent in proximal muscle groups. She denied any preceding triggers such as high‐carbohydrate meals or strenuous exercise. She also denied fever, recent gastrointestinal losses (vomiting, diarrhea), reduced oral intake, and use of diuretics or laxatives. There were no symptoms suggestive of cranial nerve involvement, sensory disturbance, or bladder or bowel dysfunction. She reported no respiratory distress, joint pain, skin rash, or oral and ocular dryness. On examination, she was alert and hemodynamically stable (blood pressure 124/78 mmHg, heart rate 86 bpm, SpO_2_: 98% on room air). Neurological examination revealed flaccid quadriparesis with a medical research council (MRC) grade of 1/5 in all limbs, absent deep tendon reflexes, and bilaterally absent plantar responses. Cranial nerve function was intact, and sensory and cerebellar examinations were unremarkable.

## 3 Differential Diagnosis, Investigations, and Treatment

Initial laboratory studies revealed severe hypokalemia and normal anion gap metabolic acidosis, prompting further workup. The key findings are summarized in Table 1.

**TABLE 1:** Laboratory investigations revealing hypokalemia and normal anion gap metabolic acidosis.

Test	Patient value	Reference range
Serum potassium	1.4 mmol/L	3.5–5.0 mmol/L
Serum sodium	142 mmol/L	135–145 mmol/L
Serum chloride	127 mmol/L	98–107 mmol/L
Serum bicarbonate	9 mmol/L	22–28 mmol/L
Serum urea	39 mg/dL	15–45 mg/dL
Serum creatinine	0.79 mg/dL	0.6–1.2 mg/dL
Serum uric acid	2.19 mg/dL	3.4–7.0 mg/dL
Arterial blood gas—pH	7.17	7.35–7.45
Arterial pCO_2_	27.2 mmHg	35–45 mmHg
ABG HCO_3_^−^	9 mmol/L	22–28 mmol/L
Anion gap	6	8–12
TSH	0.72 μIU/mL	0.4–4.5 μIU/mL
Free T3/Free T4	2.1 pg/mL/1.2 ng/dL	Normal
Vitamin B12	196 pg/mL	Low‐normal (200–900 pg/mL)

*Note:* Laboratory results demonstrated profound hypokalemia and non‐anion gap metabolic acidosis with preserved renal function. Arterial blood gas (ABG) confirmed a pH of 7.17, a bicarbonate of 9 mmol/L, and an anion gap of 6, consistent with dRTA. Thyroid‐stimulating hormone and free T3/T4 levels were within normal limits, and vitamin B12 was borderline low. The borderline low vitamin B12 level was noted but considered an incidental finding, unlikely to be related to the acute presentation.

Abbreviations: ABG, arterial blood gas; AG, anion gap; dRTA, distal renal tubular acidosis; HCO_3_^−^, bicarbonate; TSH, thyroid‐stimulating hormone; T3, triiodothyronine; T4, thyroxine.

**FIGURE 1:**
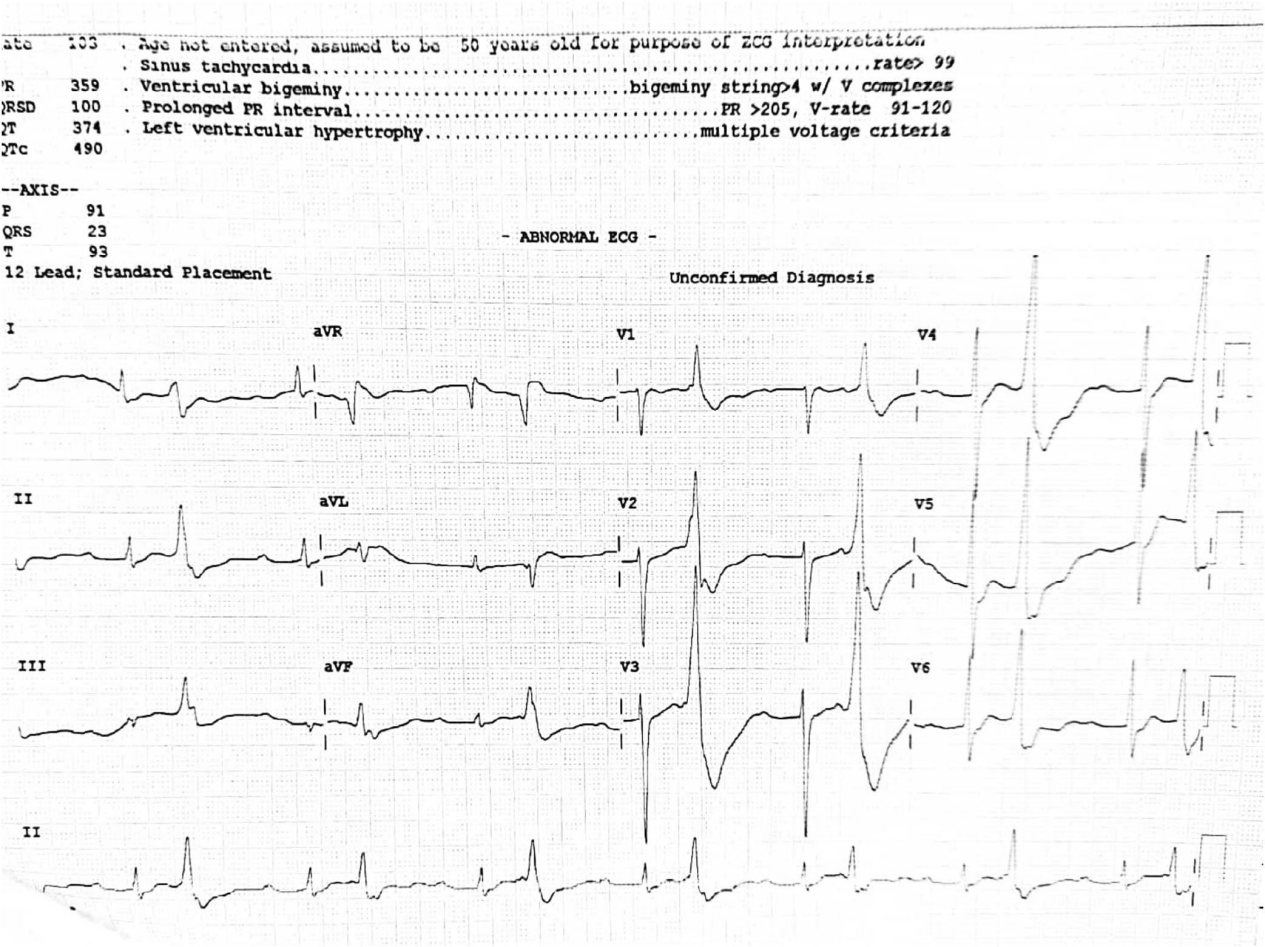
ECG at presentation demonstrating arrhythmic and repolarization abnormalities of severe hypokalemia.

The ECG (Figure 1) demonstrated sinus tachycardia with frequent ventricular ectopy in a bigeminy pattern. Conduction abnormalities included first‐degree atrioventricular block (PR interval > 200 ms). Voltage criteria for left ventricular hypertrophy were met. Repolarization changes consistent with severe hypokalemia were evident, including ST segment depression, T wave flattening/inversion, prominent U waves in precordial leads, and a prolonged QTc interval (~490 ms).

A renal ultrasound was performed, which was normal and showed no nephrolithiasis or nephrocalcinosis. Urinary studies were performed to evaluate dRTA (Table 2). A urine pH of 6.7 despite systemic acidosis, coupled with hypokalemia and elevated urinary potassium excretion, supported the diagnosis.

**TABLE 2:** Urine studies in suspected dRTA.

Test	Result	Reference range
Urine pH	6.7	Normally < 5.5 in acidosis
24 h Urine potassium	33 mmol/day	< 20 mmol/day (in hypokalemia)
24 h Urine sodium	60 mmol/day	Variable
Urine volume (24 h)	1.0 L	> 1.5 L
Random urine potassium	15 mmol/L	Variable
Random urine sodium	50 mmol/L	Variable

*Note:* 24‐h collection.

Further evaluation was undertaken to identify a potential underlying cause of dRTA. Possible autoimmune etiology was suspected. The autoimmune panel is summarized in Table 3.

**TABLE 3:** Autoimmune panel results.

Test	Result	Reference range/interpretation
ANA (HEp‐2)	Positive	Homogeneous nucleoplasmic pattern, 4+
ANA titer	1:640	Positive
Anti‐Ro/SSA	172 RU/mL	Positive (normal < 20 RU/mL)
Anti‐La/SSB	176 RU/mL	Positive (normal < 20 RU/mL)
Schirmer's test (both eyes)	5 mm	Abnormal (< 10 mm indicates dryness)
Rheumatoid factor	Negative	Normal

*Note:* ANA was positive with a homogeneous nucleoplasmic pattern at high titer (1:640). Strong positivity for anti‐Ro/SSA and anti‐La/SSB antibodies further supports the diagnosis of primary Sjögren's syndrome. Schirmer's test confirmed ocular dryness.

Abbreviations: ANA, antinuclear antibody; SSA, Sjögren's syndrome A; SSB, Sjögren's syndrome B.

Despite the absence of patient‐reported sicca symptoms, the objective findings fulfilled the 2016 ACR‐EULAR classification criteria for PSS. The patient achieved a score of 4, based on the presence of anti‐Ro/SSA antibodies (3 points) and an abnormal Schirmer's test (1 point), confirming the diagnosis [6].

The patient received intravenous potassium chloride and sodium bicarbonate, with cardiac monitoring.

## 4 Outcome and Follow‐Up

Over the next 48–72 h, her serum potassium levels normalized, and motor strength improved progressively. She was referred to rheumatology for initiation of immunomodulatory therapy and long‐term follow‐up of Sjögren's syndrome.

## 5 Discussion

Primary Sjögren's syndrome is a multisystem autoimmune disease commonly presenting with sicca symptoms. Renal involvement occurs in fewer than 10% of cases, with TIN being the most common pathology, often leading to dRTA. In dRTA, impaired hydrogen ion secretion by alpha‐intercalated cells in the distal nephron causes metabolic acidosis [2]. To maintain electroneutrality, the kidneys compensate by increasing potassium (K+) secretion in exchange for sodium (Na+) reabsorption, leading to renal K+ wasting and subsequent hypokalemia. Potassium wasting in dRTA may cause paralysis and cardiac arrhythmia, and dRTA may also manifest as nephrolithiasis or nephrocalcinosis [7].

Our patient's presentation was consistent with hypokalemic paralysis. The differential diagnosis includes familial periodic paralysis, thyrotoxic periodic paralysis, and secondary causes of hypokalemia, such as gastrointestinal losses or diuretic use. The absence of a family history, normal thyroid function, and no history of gastrointestinal losses or drug use strongly suggested a renal etiology. The laboratory findings of severe hypokalemia (1.4 mmol/L) with a concurrent normal anion gap metabolic acidosis and an inappropriately alkaline urine pH (6.7) were classic for dRTA.

The crucial next step was to identify the underlying cause of dRTA. Although our patient lacked the classic sicca symptoms, the presentation in a middle‐aged woman prompted an autoimmune workup. The diagnosis of PSS was not one of exclusion but was positively established based on the 2016 ACR–EULAR classification criteria [6]. The presence of high‐titer ANA, strong positivity for anti‐Ro/SSA and anti‐La/SSB antibodies, and an abnormal Schirmer's test provided definitive evidence for PSS.

Hypokalemic paralysis as the initial presentation of Sjögren's syndrome is extremely rare but has been documented in case reports, often in middle‐aged women who may lack sicca features at onset [8, 9]. These cases, similar to ours, consistently report profound hypokalemia, hyperchloremic metabolic acidosis, inappropriately alkaline urine, and rapid neurological recovery with potassium and bicarbonate replacement [10, 11, 12]. Quadriparesis in such settings may mimic neurological causes such as Guillain–Barré syndrome, myasthenia gravis, or spinal cord lesions, often delaying the correct diagnosis. As these reports and our case emphasize, the absence of dryness should not preclude the consideration of PSS when investigating unexplained dRTA [11, 13].

Management involves two key goals: immediate correction of life‐threatening electrolyte imbalances and long‐term treatment of the underlying autoimmune disease. Prompt intravenous potassium and bicarbonate replacement is critical to prevent cardiac arrhythmias and respiratory muscle failure, and it led to a complete recovery of muscle strength in our patient. Long‐term management of dRTA requires sustained oral alkali therapy (e.g., potassium citrate) to maintain normal serum bicarbonate and potassium levels, which helps prevent recurrent paralysis, nephrolithiasis, and progressive chronic kidney disease. The patient was referred to a rheumatologist for consideration of immunomodulatory therapy, such as hydroxychloroquine or other disease‐modifying agents, to manage the systemic autoimmune process and prevent further organ damage. The prognosis is generally favorable with consistent alkali replacement, although regular monitoring of renal function and electrolytes is essential.

Limitations include the absence of a salivary gland biopsy, which could have strengthened the diagnosis, though current ACR–EULAR criteria permit diagnosis based on serology and ocular testing.

This case highlights the rare but clinically significant initial presentation of primary Sjögren's syndrome as hypokalemic quadriparesis secondary to distal RTA. Although Sjögren's syndrome typically presents with sicca symptoms, clinicians should maintain a high index of suspicion for autoimmune causes in patients with unexplained hypokalemia and non‐anion‐gap metabolic acidosis, even in the absence of classical features. Early recognition and treatment of dRTA with potassium and bicarbonate supplementation can lead to rapid and complete neurological recovery. Furthermore, identifying the underlying autoimmune etiology is crucial for initiating appropriate long‐term immunomodulatory therapy and preventing additional systemic complications.

**Learning Points**

- Distal renal tubular acidosis (dRTA) can be the initial presentation of primary Sjögren's syndrome, even in the absence of sicca symptoms.
- The presence of non‐anion gap metabolic acidosis with persistent hypokalemia should prompt evaluation for renal tubular dysfunction.
- Autoimmune serology, including ANA, anti‐Ro/SSA, and anti‐La/SSB, plays a critical role in diagnosing Sjögren's syndrome in atypical presentations.

## Author Contributions

**Sagun Baral:** conceptualization, data curation, formal analysis, methodology, supervision, validation, visualization, writing – original draft, writing – review and editing. **Sushmita Bhattarai:** conceptualization, formal analysis, investigation, supervision, writing – review and editing. **Ajaya Raj Gautam:** methodology, supervision, visualization, writing – review and editing. **Prabesh Raghubanshi:** methodology, supervision, validation, writing – review and editing. **Krishna Kc:** methodology, supervision, writing – review and editing.

## Funding

The authors have nothing to report.

## Ethics Statement

Ethical approval was not required for this single‐patient case report in accordance with institutional guidelines.

## Consent

Written informed consent was obtained from the patient for publication of this case report and accompanying data.

## Conflicts of Interest

The authors declare no conflicts of interest.

## Data Availability

The data that support the findings of this study are available from the corresponding author upon reasonable request.
